# Type IV pili—a numbers game

**DOI:** 10.15252/embj.201489096

**Published:** 2014-06-25

**Authors:** Vijaykumar Karuppiah, Jeremy P Derrick

**Affiliations:** Faculty of Life Sciences, University of ManchesterManchester, UK

## Abstract

Type IV pili are long polymers located on the surface of a wide variety of bacterial cells, including the pathogen *Neisseria meningitidis*. They are responsible for a diverse range of functions, including adhesion, motility and natural transformation. In this issue of *The EMBO Journal*, Imhaus and Duménil show that two minor pilins, PilX and PilV, exert some of their effects by changing mean surface pilus number and that this modulates different pilus-dependent functions.

See also: **A-F Imhaus & G Duménil** (August 2014)

Type IV pili (TFP) are the most widespread class of pili or fimbriae found in bacteria; they have a particular importance in proteobacteria which cause human disease, such as *Neisseria meningitidis*, the causative agent of meningococcal meningitis, *Pseudomonas aeruginosa* and enteropathogenic *Escherichia coli* (Pelicic, 2008). An intriguing aspect of type IV pilus biology is how an ostensibly simple cell surface structure is able to mediate such a wide variety of functions. It is well established that TFP mediate adhesion to epithelial and endothelial cells, although the precise details of this process are still being elucidated. TFP are also capable of rapid retraction, a process which is associated with twitching motility and the ability of bacteria to migrate across solid surfaces. Additionally, TFP are associated with natural transformation, or competence: the ability to take up DNA from outside the cell. How are these various functions integrated and controlled from within the cell? We know little at present to answer these questions, but some pertinent observations from the recent paper from Imhaus and Duménil (2014) suggests a role for two minor pilins, PilX and PilV, in the process.

Type IV pili are predominantly composed of a major pilin protein which adopts a conserved fold, based on a single α-helix packed against a 4-stranded β-sheet. Pilins are expressed in a pre-pilin form, and then processed by a specialist peptidase, PilD, located in the inner membrane. The N-terminus forms a highly hydrophobic α-helix, a property that assists in locating it into the inner membrane. This process forms a pool of depolymerised pilin subunits in the inner membrane which acts as a reservoir for assembly of the pilus fibre (Fig1A). TFP are assembled by a complex of proteins which span the outer and inner membranes (Pelicic, 2008). From work in *N. meningitidis*, Pelicic, Nassif and co-workers have defined a ‘core’ set of biogenesis proteins which are essential for TFP assembly (Carbonnelle *et al*, 2006). The energy for pilus assembly in the periplasm is supplied by an assembly ATPase in the cytoplasm (PilF), linked to an inner membrane platform of integral membrane proteins (PilMNO) which, in turn, bind to the major pilin. Pilus assembly occurs in the periplasm, and the newly formed fibre is extruded through the outer membrane via a pore-forming secretin protein, PilQ. There are analogies between TFP biogenesis and the type II secretion system (T2SS), strikingly demonstrated by experiments which show that pilins from one system can be assembled into fibres by another (Cisneros *et al*, 2012). There are also extensive structural similarities between the principal biogenesis proteins from both systems. Type II secretion is thought to be powered by a ‘piston shunt’ mechanism: a pseudopilus forms in the periplasm, in a broadly analogous fashion to TFP formation, and ‘pushes’ the secreted substrate protein through the secretin gate (Korotkov *et al*, 2012).

**Figure 1:**
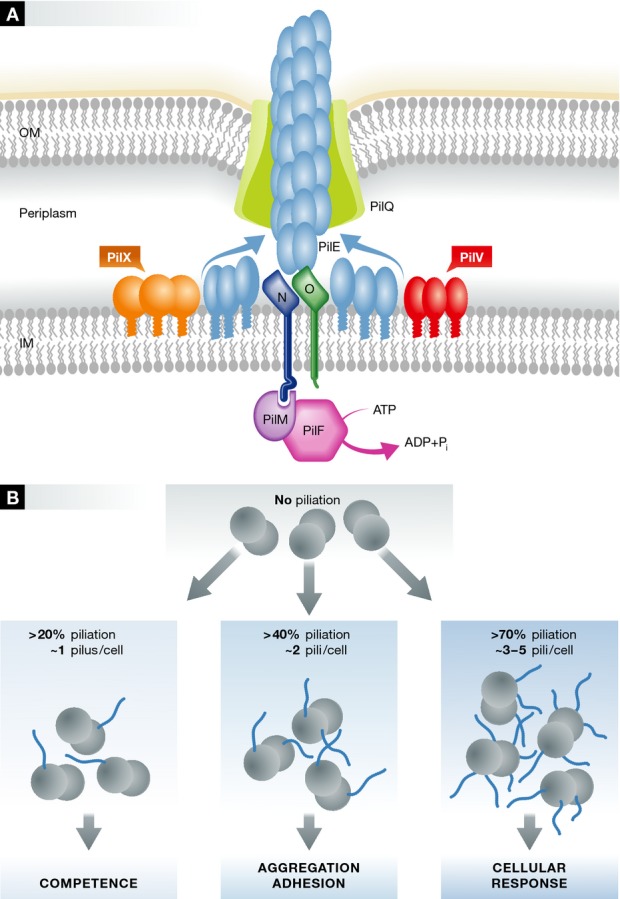
Minor pilin regulation of type IV pilus biogenesis (A) Schematic illustration of the *Neisseria meningitidis* type IV pili (TFP) biogenesis system. For clarity, some of the TFP components that are not included in the study by Imhaus and Duménil are omitted. The TFP biogenesis machine has components which are distributed from the cytoplasm to the outer membrane. The energy released from ATP hydrolysis by PilF in the cytoplasm is transferred across the inner membrane and powers the assembly of multiple subunits of the major pilin PilE. The growing fibre exits the cell via the secretin channel protein PilQ in the outer membrane. Imhaus and Duménil show that the major proportion of the minor pilins, PilX and PilV, are localised in the periplasm, rather than incorporated into the assembled fibre. (B) Modulated expression of *N*. *meningitidis*TFP surface density. The density of surface piliation was modulated by titration of the expression of the cytoplasmic PilF ATPase. At 20% piliation of the wild-type level, competence was fully restored; at 40% piliation, the bacteria were able to adhere to cells; more than 70% piliation was required to observe endothelial plasma cell membrane reorganisation.

Both TFP assembly and type II secretion are also dependent on so-called ‘minor pilins’: these are proteins which are predicted to be pilus-like in structure, but are present at much lower levels than the major pilin. Our understanding of how they might function in TFP assembly has been assisted considerably by analogy with studies on the T2SS. Formation of the T2SS pseudopilus is dependent on three pilus-like proteins, GspI, J and K. Structural studies have shown that these proteins form a complex, into an arrowhead-like structure; they are incorporated into the pseudopilus and play a critical role in initiation of pseudopilus assembly (Korotkov *et al*, 2012). In *N. meningitidis*, a similar group of minor pilins have been identified, PilH, I, J and K; mutagenesis experiments have shown that they are essential for function (Carbonnelle *et al*, 2006).

There are also other minor pilins, however, which are not essential for TFP assembly and whose roles are more poorly understood. In *N. meningitidis*, Helaine *et al* (2007) showed that PilX adopts a structure similar to that of other pilins and has a specific part of the structure—the D-region—which is essential for aggregation and adhesion. This observation was explained by Helaine *et al* through a model which involved incorporation of PilX into the assembled TFP, at a level which would permit the direct interaction of PilX with copies of the same protein on adjacent fibres in other cells. Another key property of TFP—competence—was unaffected by PilX mutation. A second minor pilin studied by Imhaus and Duménil is PilV. Mutation of PilV does not abolish TFP formation but, in common with PilX, alters TFP-associated functions. A *pilV N. meningitidis* mutant is able to aggregate and adhere to endothelial cells, but fails to elicit plasma membrane reshaping which occurs with the wild-type (Mikaty *et al*, 2009).

The puzzle, then, is how small quantities of minor pilins, apparently within the assembled TFP, are able to exert such a varied influence on the diverse biological functions? Imhaus and Duménil propose a different model, based on the effect which PilX and PilV have on the average number of TFP per cell. The heart of the paper is an elegant experiment in which they adjust the mean number of exposed pili by tuneable induction of expression of the assembly ATPase in *N. meningitidis*, PilF. The result is that they are able to modulate the level of piliation (Fig1B). They then examined three TFP-dependent properties: aggregation, adhesion to host cells and induction of a host cell response. All three properties were strikingly nonlinear with respect to the level of piliation. For example, ezrin recruitment, which was used as a marker for reorganisation of endothelial plasma cell membrane reorganisation, is virtually absent until the level of piliation exceeds half of the maximal level. On the other hand, even 20% piliation—which corresponds to one pilus per diplococcus on average—is enough to restore a full level of competence. Critically, the phenotypes associated with the *pilX* and *pilV* mutations could be reproduced by matching the mean number of pili associated with those knock-out strains. In other words, the effects of the *pilX* and *pilV* mutations can be largely explained by their indirect effects on the numbers of surface-exposed pili. Consistent with this hypothesis, they also demonstrate that only very small amounts of PilX and PilV are directly incorporated into TFP. Using imaging and biochemical experiments, the authors showed that the majority of PilV and PilX proteins are localised to and exert their properties from the periplasm (Fig1A). One such experiment involved fusing the PilV and PilX proteins with mCherry; incorporation of the fusion proteins into the pilus fibre should considerably increase its diameter, rendering the fusion proteins unlikely to exit through the secretin (PilQ) pore. As expected, PilX-mCherry and PilV-mCherry were localised in the periplasm but, critically, were still functional when expressed at wild-type levels. This indicates that the incorporation of these minor pilins into the pilus fibres is not essential, at least for the functions studied by Imhaus and Duménil. The authors therefore argue that PilV and PilX exert their effects indirectly on piliation levels through the initiation step in TFP biogenesis, although this latter conclusion will probably require further work to establish unambiguously.

The model proposed by Imhaus and Duménil is an elegant solution to some of the disparate observations which have been made through genetic mutation studies on the minor pilins. However, there are other observations which indicate that the relationship between pilus surface density, pilus structure and pilus-associated functions is more complex than it would suggest. For example, Brissac *et al* (2012) studied the effect of a *pilX* mutation on the exposure of an epitope (SM1) from the major pilin, PilE. A monoclonal antibody specific for SM1 recognises the linear epitope in pilus fibres when meningococci are bound to endothelial cells. A *pilX* mutant, however, was defective in endothelial cell signalling, and the SM1 epitope was not detected by antibody and hence not exposed in this strain. The authors suggested a role for PilX in modulating a structural transition in the pilus fibre, into a longer, narrower form: this observation has been made on the closely related TFP from *Neisseria gonorrhoeae* (Biais *et al*, 2010). In a different study, Takahashi *et al* (2012) studied the consequences of a *pilV* mutation on TFP-mediated internalisation into endothelial and epithelial cells. They observed that mutation of PilV resulted in reduced glycosylation of serine 62 in the major pilin, PilE. It may be the case that PilX and PilV exert an effect on TFP fibre structure—as well as pilus surface density—and therefore indirectly influence exposure of epitopes and potential glycosylation sites in the PilE pilin.

There are also some important, more general, ramifications of the observations of Imhaus and Duménil for the regulation of TFP-dependent functions *in vivo*. Specifically, the results suggest that modulation of piliation levels may be more subtle than has traditionally been appreciated: the nonlinearity of TFP-dependent properties with respect to piliation means that, within certain ranges, small changes in pilus number could translate into large differences in behaviour. If drugs were designed towards blocking TFP biogenesis and elicit even a 50% reduction in piliation, the effect could have a disproportionate impact on bacterial pathogenicity.
